# Efficiency of a deep learning-based artificial intelligence diagnostic system in spontaneous intracerebral hemorrhage volume measurement

**DOI:** 10.1186/s12880-021-00657-6

**Published:** 2021-08-13

**Authors:** Tao Wang, Na Song, Lingling Liu, Zichao Zhu, Bing Chen, Wenjun Yang, Zhiqiang Chen

**Affiliations:** 1 The Department of Radiology, The General Hospital of Ningxia Medical University, Yinchuan, 750004 Ningxia China; 2 Key Laboratory of Fertility Preservation and Maintenance, School of Basic Medicine and the General Hospital, Ningxia Medical University, Yinchuan, 750004 China

**Keywords:** Intracerebral hemorrhage, Spontaneous intracerebral hemorrhage, Volumetrics, Artificial intelligence, Deep learning

## Abstract

**Background:**

Accurate measurement of hemorrhage volume is critical for both the prediction of prognosis and the selection of appropriate clinical treatment after spontaneous intracerebral hemorrhage (ICH). This study aimed to evaluate the performance and accuracy of a deep learning-based automated segmentation algorithm in segmenting spontaneous intracerebral hemorrhage (ICH) volume either with or without intraventricular hemorrhage (IVH) extension. We compared this automated pipeline with two manual segmentation techniques.

**Methods:**

We retrospectively reviewed 105 patients with acute spontaneous ICH. Depending on the presence of IVH extension, patients were divided into two groups: ICH without (n = 56) and with IVH (n = 49). ICH volume of the two groups were segmented and measured using a deep learning-based artificial intelligence (AI) diagnostic system and computed tomography-based planimetry (CTP), and the ABC/2 score were used to measure hemorrhage volume in the ICH without IVH group. Correlations and agreement analyses were used to analyze the differences in volume and length of processing time among the three segmentation approaches.

**Results:**

In the ICH without IVH group, the ICH volumes measured using AI and the ABC/2 score were comparable to CTP segmentation. Strong correlations were observed among the three segmentation methods (*r* = 0.994, 0.976, 0.974; *P* < 0.001; concordance correlation coefficient [CCC] = 0.993, 0.968, 0.967). But the absolute error of the ICH volume measured by the ABC/2 score was greater than that of the algorithm (*P* < 0.05). In the ICH with IVH group, there is no significant differences were found between algorithm and CTP(*P* = 0.614). The correlation and agreement between CTP and AI were strong (*r* = 0.996, *P* < 0.001; CCC = 0.996). The AI segmentation took a significantly shorter amount of time than CTP (*P* < 0.001), but was slightly longer than ABC/2 score technique (*P* = 0.002).

**Conclusions:**

The deep learning-based AI diagnostic system accurately quantified volumes of acute spontaneous ICH with high fidelity and greater efficiency compared to the CTP measurement and more accurately than the ABC/2 scores. We believe this is a promising tool to help physicians achieve precise ICH quantification in practice.

**Supplementary Information:**

The online version contains supplementary material available at 10.1186/s12880-021-00657-6.

## Background

Spontaneous intracerebral hemorrhage (ICH) is a severe medical concern and one of the leading causes of morbidity and mortality worldwide [1, 2]. The annual overall incidence rate is approximately 24.6 cases per 100,000 people, and the median case fatality was approximately 40.4% during the first month of illness [3]. Moreover, hemorrhage volume is a powerful predictor of the 30-day prognosis following diagnosis [4]. Hematoma expansion and the presence of intraventricular hemorrhage (IVH) extension are closely correlated to poor outcomes in patients with ICH [5–7]. Recent clinical trials investigating new ICH treatments have included hematoma volume as one of the eligibility criteria to determine which patients are best suited for intervention [8, 9]. Accordingly, hematoma volume is not only one of the indicators of the patient’s prognostic score but also an important marker of clinical treatment options. Therefore, quick and accurate measurements of ICH volume have become clinically essential. Computed tomography (CT)-based planimetry (CTP) and ABC/2 score are the two primary manual methods used for the measurement of ICH volume in clinical practice and research [2, 9, 10]; however, CTP is time consuming and the accuracy of the ABC/2 score decreases with large, irregular, or lobar hematoma [11–13].

To improve the efficiency of quantitative ICH, research has presented various methods based on machine learning for the automatic segmentation of ICH. Machine learning is a subset of artificial intelligence (AI) that is widely used in many fields [14]. Deep learning is a recently developed machine learning technology that simulates the human brain with multiple layers of artificial neural networks [14, 15]. Traditional computer vision techniques are based on handcrafted features; however, deep learning models are learned and extracted automatically [16]. Accordingly, its network can learn by analyzing the training data and make predictions as new data are entered, requiring little manual engineering [15, 17]. Some deep learning-based AI diagnosis systems have been developed to detect and segment cerebral hemorrhage,[18, 19] but the accuracy of these algorithms and their practical clinical value require further external verification. IVH has been associated with mortality rates as high as 50–75% [20, 21] and increasing the accuracy of the definitions of IVH volume could improve its ability to predict outcomes.[22, 23] However, the presence of IVH may blur the boundaries between intraparenchymal and intraventricular blood, and consequently, it’s challenging to accurately differentiate IVH from the adjacent parenchymal hematoma, even for experienced human raters [18, 24, 25]. Researchers have tried different techniques to find a more efficient way to define hematoma expansion including IVH expansion. By detecting the ICH volume of patients without and with IVH using CTP, David et al*.* [25] demonstrated that the minimal detectable difference for total ICH hemorrhage volume measurement was greater in the presence of IVH. To our knowledge, however, there are few studies that have examined the difference in the accuracy of deep learning-based diagnosis systems in segmenting ICH volume, either with or without IVH extension. In the present study, patients with acute spontaneous ICH were divided into two groups based on the presence of IVH extension: ICH without and with IVH groups. By measuring the total hemorrhage volume, we aimed to evaluate the performance of a deep learning-based automated segmented algorithm and compared it with two manual segmentation techniques: CTP and ABC/2 score.

## Methods

### Patient selection and grouping

We retrospectively collected patient data from those who were initially diagnosed with ICH in the General Hospital of Ningxia Medical University, China, between July 2017 and December 2018. Patients with trauma, vascular abnormalities, brain tumors, hemorrhagic transformation after cerebral infarction, or any other secondary causes of ICH were excluded. A total of 105 patients with acute spontaneous ICH (within 72 h of manifestation of clinical symptoms) were included in the current study. They were divided into two groups: ICH without IVH (56 patients) and ICH with IVH (49 patients).

### Quantification of ICH

Hemorrhage volume measurements of the two groups were conducted using deep learning-based automated segmentation algorithm and CTP segmentation techniques, and the ABC/2 scores were used to measure hemorrhage volume in the ICH without IVH group. Additional file 1: Fig. S1 shows the segmentation examples of hematoma using different segmentation techniques.

#### Deep learning-based automated segmentation algorithm

The InferRead CT Stroke (InferVision, Beijing, China) is a standardized and fully automated computer-aided diagnostic (CAD) system of stroke diseases and was used as the AI diagnostic system. Specifically, the segmentation of hemorrhage lesions is one of the main functions of this AI system. Using 3000 head CT scans from multi-centers as well as multi-scanners and implementing deep convolutional neural networks, refined from Dense-Net and U-Net architecture, the hemorrhage segmentation model was developed. In an internal test containing a dataset of 71 cases of ICH, the model achieved an accuracy of 0.99, a Dice similarity coefficient of 0.89, and an F1 score of 0.89.

#### Manual segmentation techniques

Hematoma volume was first measured with CTP by two independent raters (Drs. Z Chen and T Wang, neuroradiologists specialized in stroke and did not participate in the training phase of the deep learning algorithm) blinded to the results of the algorithm segmentation. They used the Extended Brilliance Workstation (Philips, Amsterdam, The Netherlands) to segment the hematoma area (including intraparenchymal hemorrhage and IVH volume, excluding subarachnoid hemorrhage) slice-by-slice for hematoma area calculation. The volume of hematoma was calculated as follows: V = Σ hematoma area × slice thickness, with measurements in milliliters. The average of hematoma volume measured by the two raters was finally used as the ground-truth volume.

The hematoma volume of the ICH without IVH group was also independently measured using the ABC/2 score by the two raters. In this formula, A is the maximum length on the slice with the largest clot area, B is the maximum width perpendicular to A on the same slice, and the number of slices containing the hematoma were then multiplied by the slice thickness to yield C. The hematoma volume was then calculated and converted into milliliters. The mean of the bleeding volume, measured by the two raters, was used for the final statistical analysis.

Finally, the time to complete each volumetric analysis time was recorded separately to compare the measurement speed.

### Statistical analysis

Baseline demographics between the two groups of patients were compared using Pearson’s or Fisher’s exact tests for categorical data, and the *t* or Mann–Whitney *U* tests for continuous data, as appropriate. ICH volume and calculation time were analyzed using standard descriptive statistics. Agreement between the two radiologists and different measurement approaches were assessed using intraclass correlation coefficient (ICC) and concordance correlation coefficient (CCC), respectively. Pairwise correlations among ICH volumes measured from each of the three segmentation methods were assessed using the Pearson correlation coefficient. Meanwhile, limits of agreement (LOA) were calculated and Bland–Altman plots were drawn to assess agreement between different measurement methods [26]. The Friedman test or Wilcoxon signed-rank test was used to analyze the differences in volume and calculation time among the three hematoma segmentation methods. *P* values of < 0.05 were considered significant. All statistical analyses were performed using Stata (version 15.0; StataCorp, College Station, TX, USA), MedCalc (version 15.6.1; MedCalc Software, Ostend, Belgium) and PRISM software (version 8.0.1; GraphPad Software Inc., San Diego, CA, USA).

## Results

### Demographic data

We included 56 patients in the ICH without IVH and 49 patients in the ICH with IVH groups. The two groups did not show significant differences in age, gender, or hematoma location (*P* > 0.05). More detailed information is shown in Table 1.

**Table 1:** Comparison of the demographics and volumetry between two groups of patients

	ICH without IVH group (n = 56)	ICH with IVH group (n = 49)	*P* value
Age, years (mean ± SD)	56.6 ± 14.7	56.4 ± 14.7	0.299^*^
Male patients (n, %)	36 (64%)	28 (57%)	0.454^†^
Deep location (n, %)	39 (70%)	39 (80%)	0.245^†^
Lobar location (n, %)	10 (18%)	8 (16%)	0.836^†^
Infratentorial location (n, %)	7 (12%)	2 (4%)	0.170^‡^
Oral anticoagulants(n,%)	1 (2%)	1 (2%)	–
*Deep learning-based automated algorithm segmentation*			
Mean volume, ml (± SD)	20.60 ± 19.17	51.74 ± 39.57	< 0.001^*^
Media volume, ml	16.43	40.32	
Range (min, max)	0.33, 96.20	3.53, 170.0	
IQR, ml	6.89, 28.49	25.80, 72.90	
Measure time, min (± SD)	1.02 ± 0.19	0.95 ± 0.09	0.021^*^
*CTP (ground-truth)*			
Mean volume, ml (± SD)	20.69 ± 18.76	51.63 ± 39.84	< 0.001^*^
Media volume, ml	15.69	40.99	
Range (min, max)	0.73, 91.07	3.33, 172.79	
IQR, ml	6.30, 29.93	24.36, 68.66	
Measure time, min (± SD)	6.98 ± 5.11	15.22 ± 7.04	< 0.001^*^
*ABC/2 score*			
Mean volume, ml (± SD)	22.22 ± 20.89	–	–
Media volume, ml	15.97	–	
Range (min, max)	0.54,89.52	–	
IQR, ml	6.84,32.31	–	
Measure time, min (± SD)	0.84 ± 0.20	–	–

ICH indicates intracerebral hemorrhage; and IVH, intraventricular hemorrhage; CTP indicates CT-based planimetry; IQR, interquartile range

*Mann–Whitney test

^†^Pearson Chi-square tests

^‡^Fisher exact test

### ICH quantification

In the ICH without IVH group, ICC between the two independent raters indicated excellent interrater agreement for manually measured data (CTP: ICC = 0.979, 95% confidence interval [CI]: 0.965 to 0.988; ABC/2 score: ICC = 0.988, 95% CI: 0.979 to 0.993). In the ICH with IVH group, agreement between the two raters was also strong (CTP; ICC = 0.983, 95% CI: 0.971 to 0.991; see Additional file 1: Table S1). Therefore, the CTP segmented ICH volume was regarded as the ground-truth volume for further analysis.

Results from the ICH quantification for the two groups are shown in Table 1. In the ICH without IVH group, the differences in the measured ICH volume between CTP and the algorithm or ABC/2 score were not significant (*P* = 0.218 and *P* = 0.658, respectively, Table 2 and Additional file 1: Fig. S2). In the ICH with IVH group, the difference in the measured ICH volume between CTP and algorithm was not significant (*P* = 0.941; Table 2 and Additional file 1: Fig. S2).

**Table 2:** Agreement comparisons among different segmentation methods for spontaneous ICH volume in two groups

Agreement Statistics	ICH without IVH group			ICH with IVH group
	Algorithm versus CTP	ABC/2 score versus CTP	Algorithm versus ABC/2 score	Algorithm versus CTP
*Difference, mL*				
Range (min, max)	− 7.00,7.670	− 8.77,21.35	− 22.04, 7.59	− 14.02,10.80
Mean	− 0.10	1.53	− 1.63	− 0.11
Median	− 0.30	− 0.33	− 0.60	− 0.03
IQR	− 0.77,0.47	− 0.50,2.25	− 2.51,0.54	− 0.94,1.43
95% LOA (low, high)	− 4.38,4.18	− 7.90,10.96	− 11.22,7.96	− 7.05,6.82
CCC [95% CI]	0.993 [0.989 to 0.996]	0.968 [0.948 to 0.980]	0.967 [0.946 to 0.980]	0.996 [0.993 to 0.998]
*P*	0.218^*^	0.658^*^	0.007^*^	0.941^†^

ICH indicates intracerebral hemorrhage; IVH, intraventricular hemorrhage; CTP, CT− based planimetry; LOA, limits of agreement; IQR, interquartile range; CCC, concordance correlation coefficient; and CI, confidence limit

*Friedman test, followed by pairwise comparisons

^†^Wilcoxon signed−rank test

**Table 3:** Pairwise comparisons of differences in volumetric analysis times among segmentation methods in two groups

Statistics	ICH without IVH group			ICH with IVH group
	Algorithm versus CTP	ABC/2 score versus CTP	Algorithm versus ABC/2 score	Algorithm versus CTP
*Difference, min*				
Range (min, max)	− 25.12,− 0.07	− 25.17,− 0.37	− 1.08,− 0.37	− 31.05,− 1.12
Mean	− 5.96	− 6.15	0.18	15.22
Median	− 4.51	− 4.66	− 0.16	− 13.97
IQR	− 7.88,− 2.17	− 8.03,− 2.47	− 0.28,− 0.03	− 18.97.− 8.64
*P*	< 0.001*	< 0.001*	0.002*	< 0.001^†^

ICH indicates intracerebral hemorrhage; IVH, intraventricular hemorrhage; CTP, CT− based planimetry; IQR, interquartile range

*Friedman test, followed by pairwise comparison

^†^ Wilcoxon signed− rank test

### Correlation and agreement analysis

**Fig. 1:**
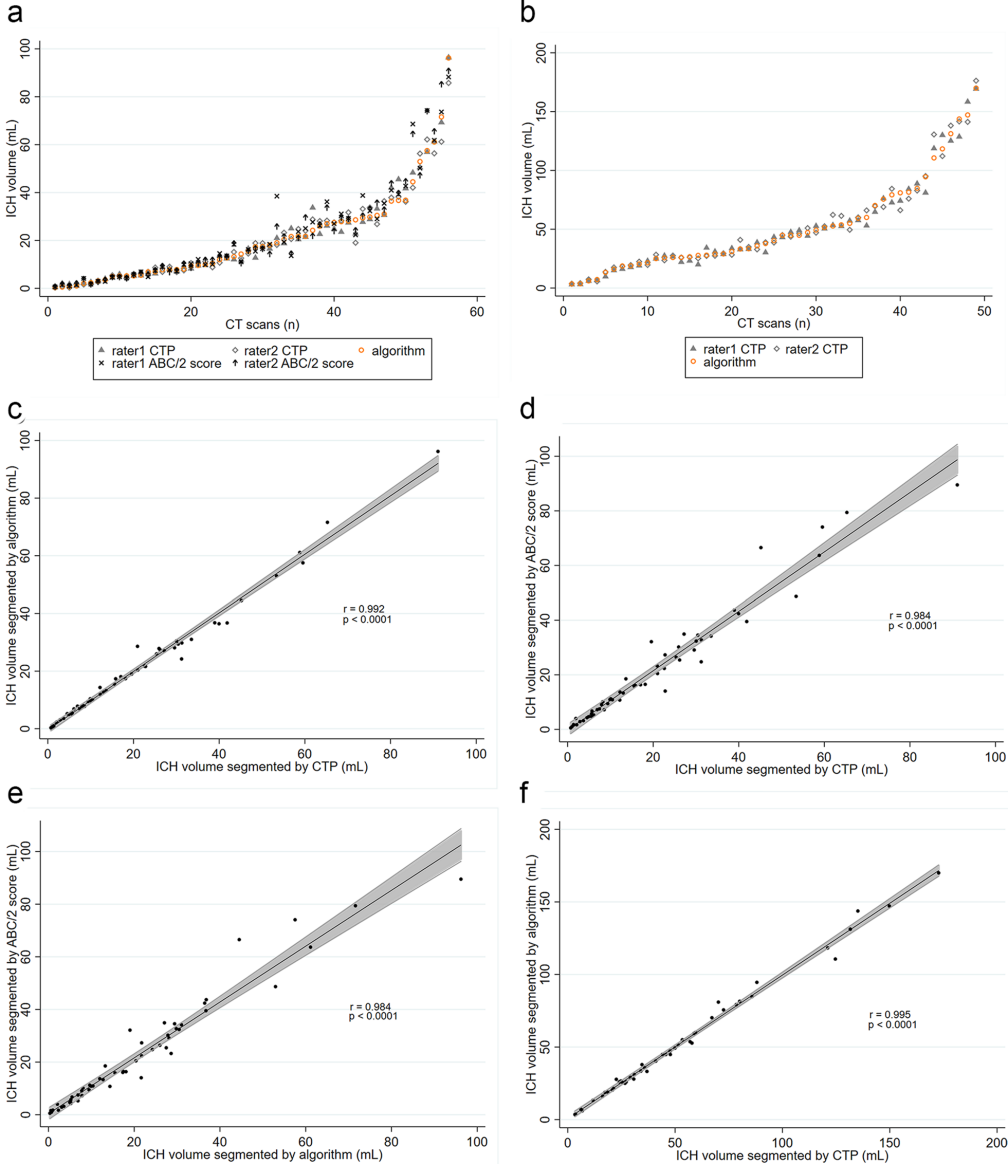
Scatter plots comparing segmented spontaneous intracerebral hemorrhage (ICH) volumes among the algorithm, ABC/2 score and CTP segmentation methods. **a**, **b** Comparison of the segmented ICH volumes for each user between different segmentation methods in ICH without intraventricular hemorrhage (IVH) group (**a**), and ICH with IVH group (**b**). **c**–**f**, Comparison of segmented ICH volumes among both user for algorithm versus CTP (**c**) ABC/2 score versus CTP (**d**) algorithm versus ABC/2 score (**e**) in ICH without IVH group, and algorithm versus CTP (**f**) segmentation methods in ICH with IVH group

Figure 1a, b demonstrate the segmented ICH volumes by different raters and segmentation methods in each group. In the ICH without IVH group, strong correlations were observed among the three segmentation methods (*r* = 0.994, 0.976, 0.974, *P* < 0.001; Fig. 1c–e). In the ICH with IVH group, strong correlations were also found between the CTP and algorithm (*r* = 0.996, *P* < 0.001; Fig. 1f).

**Fig. 2:**
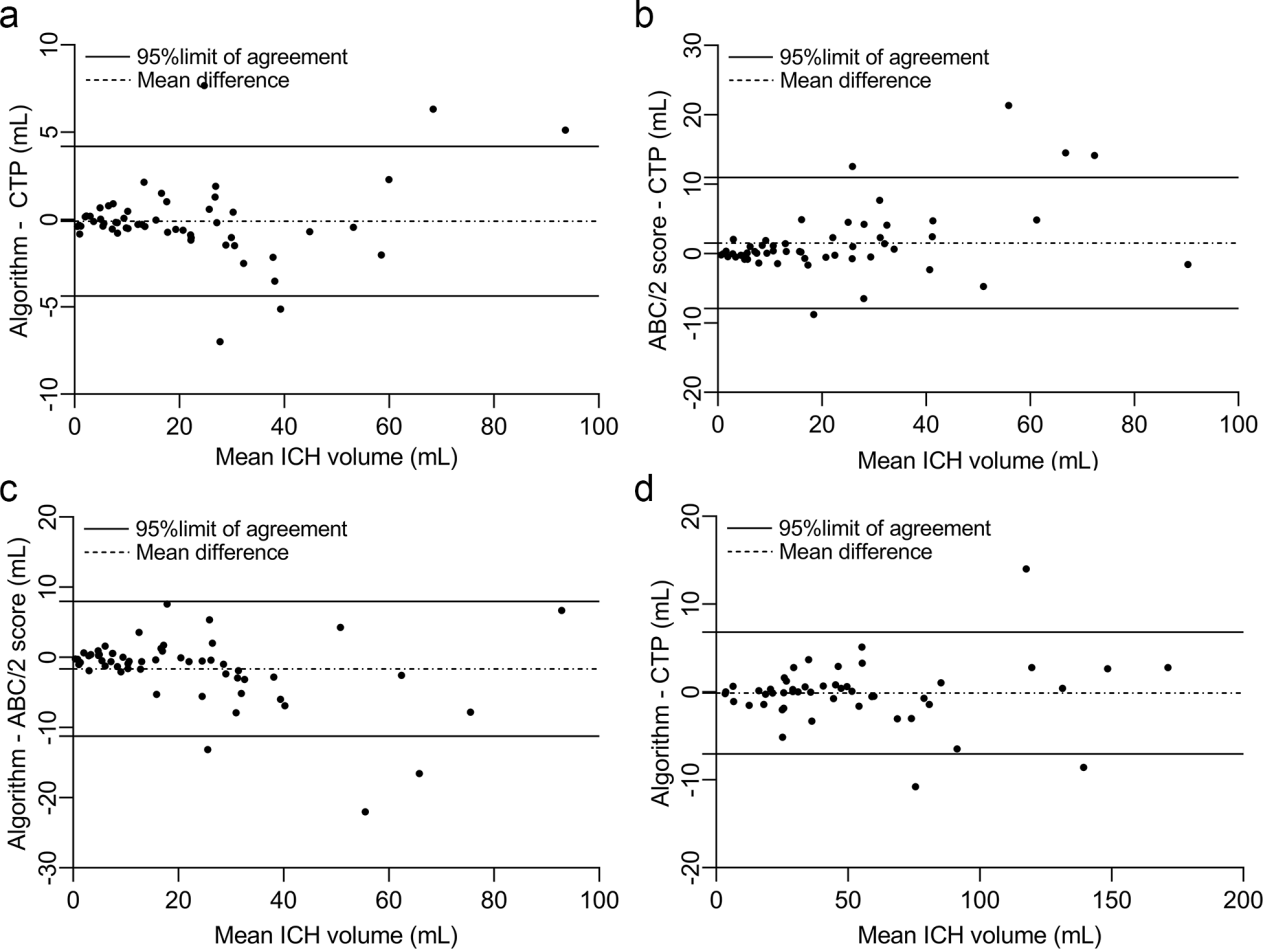
Agreement analysis of spontaneous intracerebral hemorrhage (ICH) volume segmented by different methods in two groups. **a**–**c**, Agreement illustrated for algorithm versus CTP (**a**), ABC/2 versus CTP (**b**), and algorithm versus ABC/2 score (**c**) segmentation methods in ICH without intraventricular hemorrhage (IVH) group, and algorithm versus CTP (**d**) segmentation methods in ICH with IVH group

**Fig. 3:**
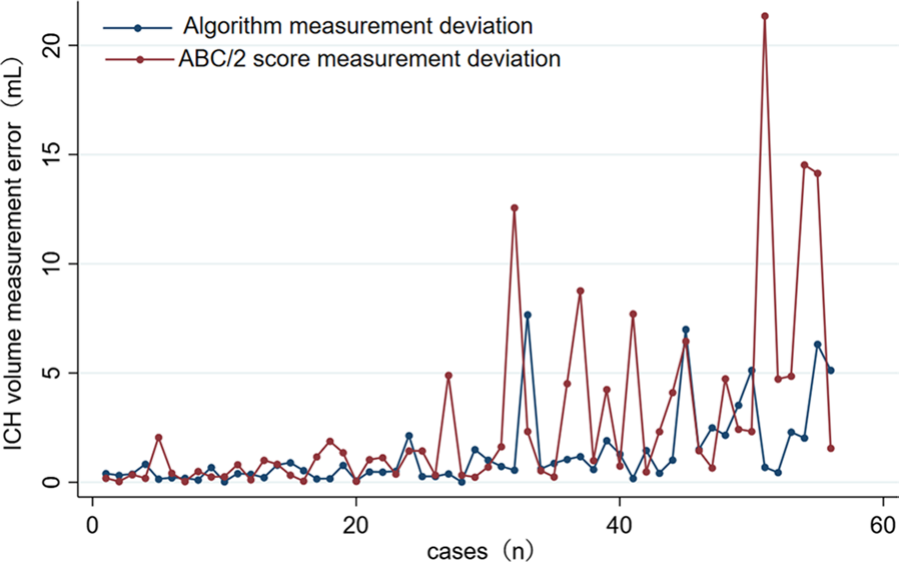
The difference curve of absolute error of the ICH volume measured by the ABC/2 score and algorithm in intracerebral hemorrhage without intraventricular hemorrhage group

In the ICH without IVH group, strong agreement, among the three different segmentation methods, was illustrated with CCC and Bland–Altman analyses (Fig. 2a–c and Table 2). The mean deviation values were − 0.10 for the algorithm versus CTP, 1.53 for ABC/2 score versus CTP, and − 1.63 for algorithm versus ABC/2 score. The 95% LOA values were − 4.38 to 4.18, 10.96 to − 7.90, and − 11.22 to 7.96 mL, respectively. The difference curve showed that the absolute error of the ICH volume measured by the ABC/2 score was greater than that of the algorithm (*P* < 0.05, Fig. 3).

In the ICH with IVH group, agreement between the algorithm and CTP was strongly demonstrated by the mean deviation of 0.11 mL, 95% LOA of − 6.82 to 7.05 mL (Fig. 2d), and CCC of 0.996 (0.993 to 0.998; Table 2).

### Volumetric analysis time

The volumetric analysis times of the different segmentation methods for the two groups are shown in Table 1. The volumetric analysis time of the algorithm was significantly shorter than the CTP segmentation method in both the groups (median differences were − 4.51 [− 7.88 to − 2.17] min/scan for the ICH without IVH group and − 13.97 [− 18.97 to − 8.64] min/scan for the ICH with IVH group; *P* < 0.001; Table 3). Further, the volumetric analysis time of the algorithm was slightly longer than the ABC/2 score method (median difference = − 0.16 [− 0.28 to − 0.03] s/scan; *P* = 0.002, Table 3).

## Discussion

In the present study, we evaluated the efficacy of a deep learning-based AI diagnostic system in measuring the total volume of acute spontaneous ICH by comparing this method with two other manual volume segmentation techniques. Our agreement analysis of ICH volume showed that AI was comparable to CTP segmentation in patients either with or without the presence of IVH extension. Further, the agreement analysis showed that the ABC/2 score was comparable to the CTP in patients without IVH extension, but its absolute measurement error was greater than the algorithm segmentation. Moreover, the average time of the AI system model, which was slightly longer than the ABC/2 score, took less than one tenth of the time of CTP.

The deep learning algorithm labels a target with a pixel-wise precise boundary and segments it.The volume of each pixel was calculated by combining CT slice thickness, and the hemorrhage volume was calculated by accumulating all the volumes of pixels in the hemorrhage region [19].

Several different deep learning models have been developed for ICH automated quantification. Research has supported that the volume of ICH segmented by deep learning models, which is faster than manual CTP, yields similar results to CTP; [18, 19, 28–30]this is consistent with our present results.

A group of manual methods, including CTP and the ABC/2 score, have long been available for measuring ICH volume. The ABC/2 score is based on a simplified ellipsoid volumetric formula.[4, 10] Many previous studies have found that ICH volume measured using the ABC/2 score is highly correlated with CTP measurement, and volumetric analysis time was less than 1 min per case.[4, 12, 31] Therefore, the ABC/2 score has been commonly used in clinical settings and research to rapidly quantify the volume of ICH. Our current analysis demonstrated that the accuracy of the deep learning-based AI model was the same as that of the ABC/2 score in segmenting ICH volume without IVH extension. Several studies reported that the measurement errors of the ABC/2 score increased with larger ICH hematoma and clot irregularity [11, 12, 32–34]. We also observed that the ABC/2 score overestimated some ICH volumes when confronting larger and irregular morphology features (Additional file 1: Fig. S3).

Various studies have shown that deep learning also performs well in detecting and measuring the midline shift [35–37]. Accordingly, we also extracted midline shift data (maximum septum pellucidum shift), measured by the algorithm and radiologist (reference standard), from 73 patients with spontaneous supratentorial ICH out of all the patients enrolled. A strong correlation and agreement was observed between the algorithm and reference standard (*r* = 0.858, *P* < 0.001; ICC = 0.853 [0.775 to 0.905]; Additional file 1: Fig. S4a and Additional file 1: Table S2) for midline shift measurement. Consistent with previous reports, our results revealed that the algorithm showed potential advantages in the evaluation of the midline shift.

Our study has some limitations. First, random sample selection does not balance different hematoma locations or morphology features, which may negatively influence the accuracy of ABC/2 in the agreement analysis. Second, we used the agreement analysis and Bland–Altman plots to compare the volumetric results among different methods. The dice coefficient, however, which can compare not only the volumetric results, but also the clot shape between any two methods, should be considered in future studies. Third, hypodensities on noncontrast CT were reported to be frequently present in patients with oral anticoagulants [38], which may affect accuracy of ICH volume segmentation results using the algorithm. There were only two cases with oral anticoagulants in our study, and more research work focusing on this subject is warranted in the future. Lastly, it would be interesting and meaningful to retrieve ICH and IVH volumes separately to specify volumetric analysis with regards to its prognostic significance in ICH. More practical and effective function, including this promising selective segmentation technique in the algorithm system of AI is still anticipated.

## Conclusion

In summary, the deep learning-based AI diagnostic system accurately quantified volumes of acute spontaneous ICH with high fidelity and greater efficiency compared to the CTP measurement and more accurately than the ABC/2 scores. We believe this is a promising tool to help physicians achieve precise ICH quantification in practice.

Further research is needed to determine the application of AI for hematoma volume measurement in a larger sample size or in other types as well as different phases of cerebral hemorrhage.

## Supplementary Information

**Additional file 1.** Supplementary tables and figures.

## Data Availability

All data generated or analyzed during this study are included in this published article [and its supplementary information file].

## Abbreviations

ICH: Intracerebral hemorrhage
IVH: Intraventricular hemorrhage
AI: Artificial intelligence
CTP: Computed tomography-based planimetry
CAD: Computer-aided diagnostic
ICC: Intraclass correlation coefficient
CCC: Concordance correlation coefficient
LOA: Limits of agreement
