# Supplementary material for: Efficiency of a deep learning-based artificial intelligence diagnostic system in spontaneous intracerebral hemorrhage volume measurement
Source: BMC Med Imaging. 2021 Aug 13;21:125. doi: 10.1186/s12880-021-00657-6 (PMC8364089; doi:10.1186/s12880-021-00657-6)
Supplement: Supplementary file 1 — Additional file 1. Supplementary tables and figures. [file 12880_2021_657_MOESM1_ESM.doc]

| Table S1. Agreement analysis of spontaneous ICH volume measured by two raters in two groups | | | |
| --- | --- | --- | --- |
| Agreement statistics | ICH without IVH group | | ICH with IVH group |
|  | rater1 vs. rater 2 (CTP) | rater1 vs. rater 2 (ABC/2 score) | rater1 vs. rater 2  (CTP) |
| Difference, ml |  |  |  |
| Range (min, max) | -6.12,10.62 | -11.51,12.72 | -14.12,17.88 |
| Mean | 0.08 | 0.22 | -1.06 |
| Media | -0.53 | 0.09 | -1.78 |
| IQR | -2.57, 2.17 | -0.83,1.57 | -5.94,4.34 |
| ICC [95% CI] | 0.979 [0.965 to 0.988] | 0.988 [0.979 to 0.993] | 0.983 [0.971 to 0.991] |
| ICH indicates intracerebral hemorrhage; IVH, intraventricular hemorrhage; CTP, CT-based planimetry; IQR, interquartile range; ICC, intraclass correlation coefficient; and CI, confidence limit. | | | |

| Table S2 Agreement analysis of spontaneous ICH midline shift measured by different raters and methods. | | |
| --- | --- | --- |
| Agreement statistics | rater1 vs. rater 2 | Algorithm vs. reference standard |
| Difference, mm |  |  |
| Range (min, max) | -3.10 to 2.10 | -3.89 to 3.65 |
| Mean | -0.06 | -0.34 |
| Media | 0 | 0 |
| IQR | -0.15, 0.15 | -2.10,0.05 |
| ICC [95% CI] | 0.981 [0.970 to 0.988] | 0.853 [0.775 to 0.905] |
| *P** | 0.925 | 0.145 |
| IQR indicates interquartile range; ICC, intraclass correlation coefficient; CI, confidence limit.  *** Wilcoxon signed-rank test. | | |


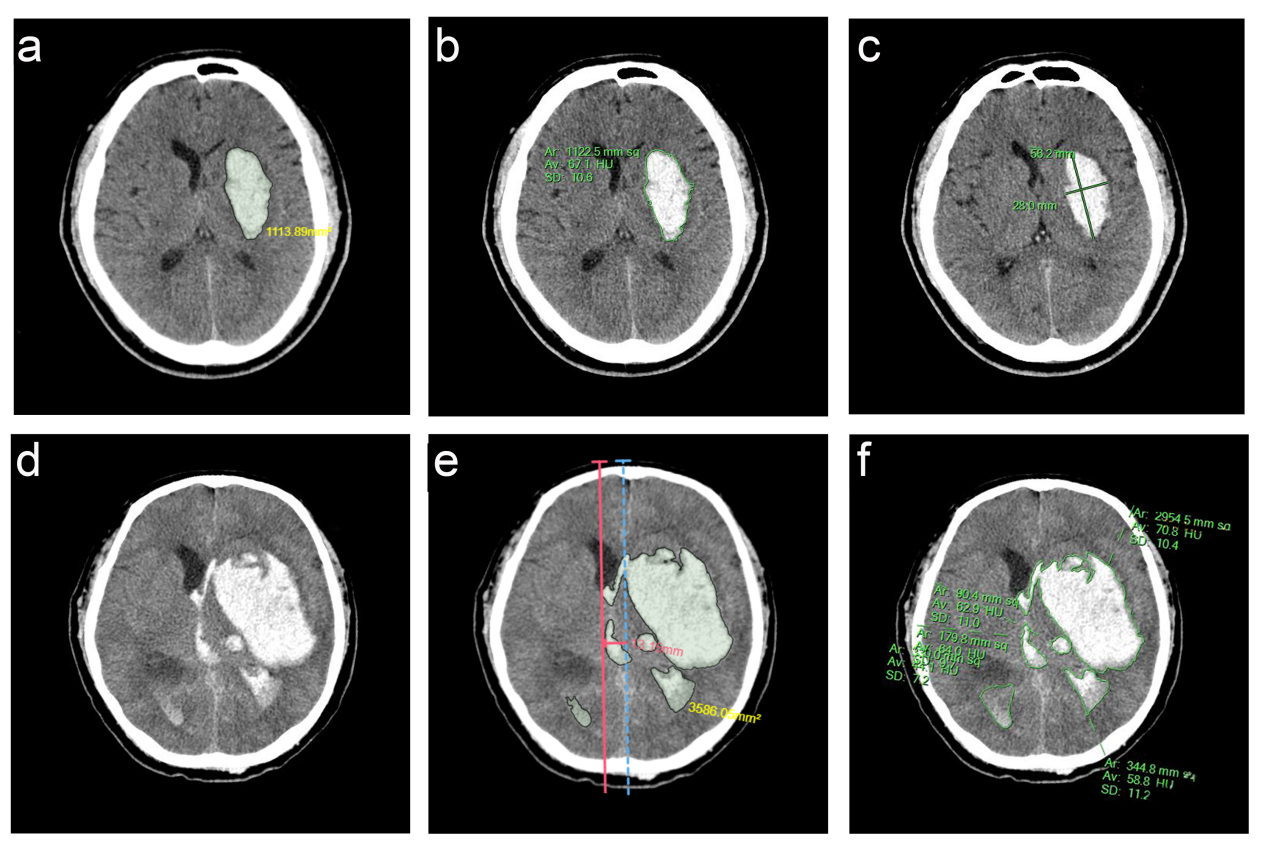
Fig. S1 Examples of intracerebral hemorrhage (ICH) segmentation in two groups. a-c, depicts the algorithm segmentation (a), CTP segmentation (b) and the corresponding ABC/2 score diameter measurement (c) in ICH without intraventricular hemorrhage (IVH) group. d-f depicts the original CT slice image (d), the corresponding algorithm segmentation (e) and CTP segmentation (f) in ICH with IVH group.


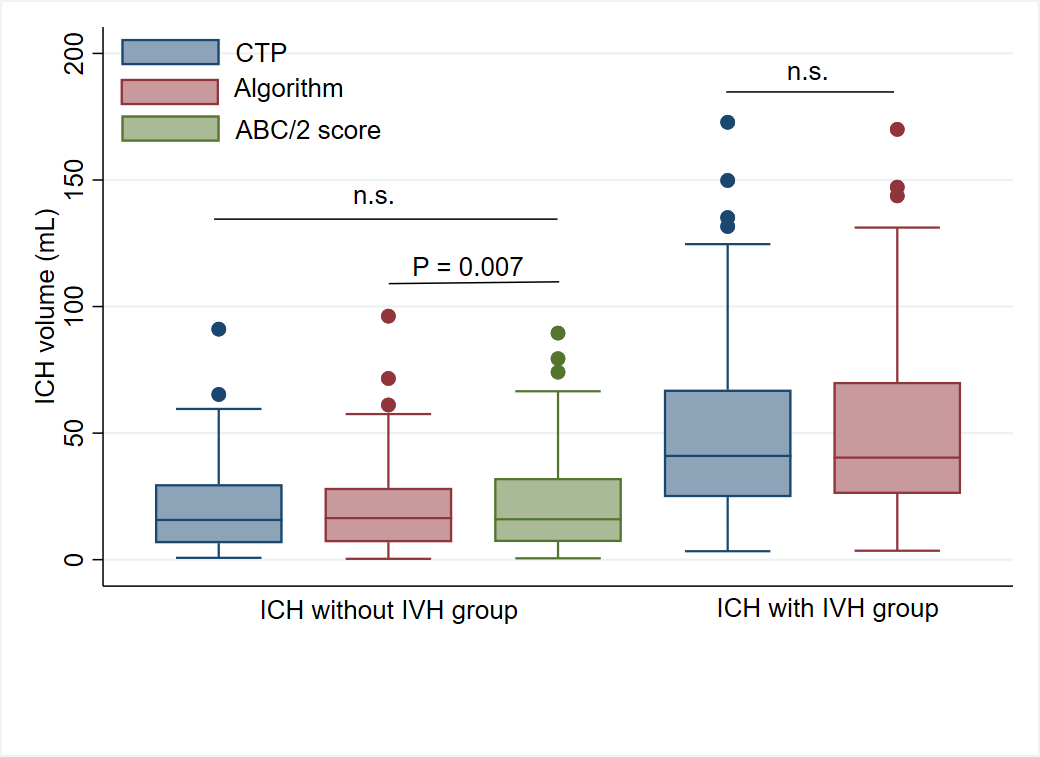
Fig. S2 Mean intracerebral hemorrhage (ICH) volumes retrieved by CTP, algorithm segmentation and ABC/2 score in two groups. No significant difference was observed between the CTP and algorithm or ABC/2 score (*P >* 0.05 Friedman test, followed by followed by pairwise comparisons). significant differences were observed only for ABC/2 score compared with the algorithm segmentations. n.s. indicates not significant (*P* > 0.05).


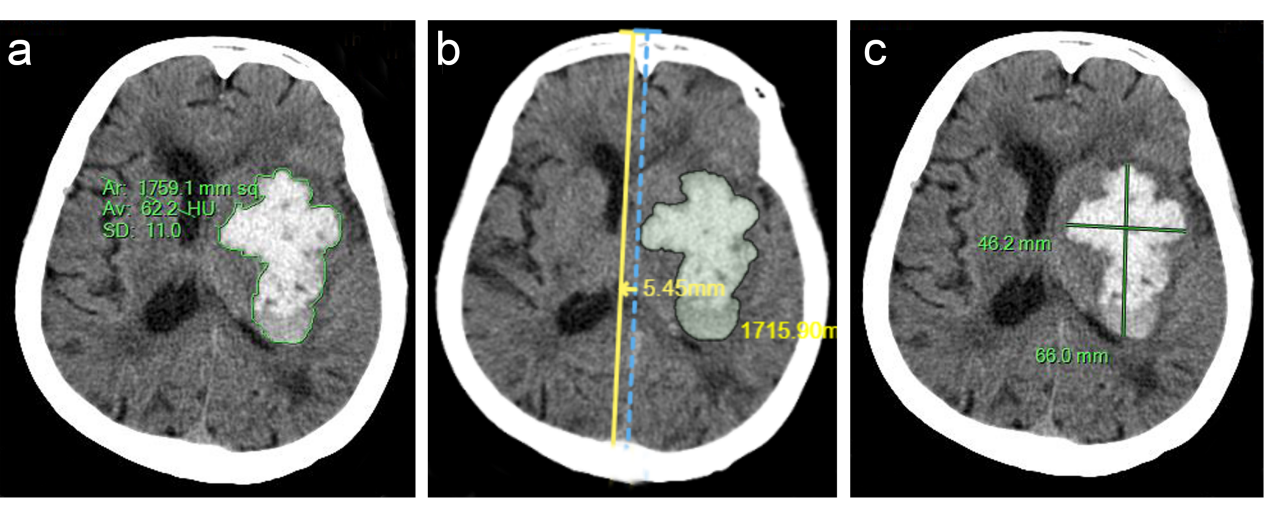


Fig. S3 Hemorrhage volume of an ICH patient with irregular hematoma (Barras grade V) segmented by CTP (a), algorithm (b) and ABC/2 score (c) were 45.18mL, 44.49mL and 66.53mL, respectively.


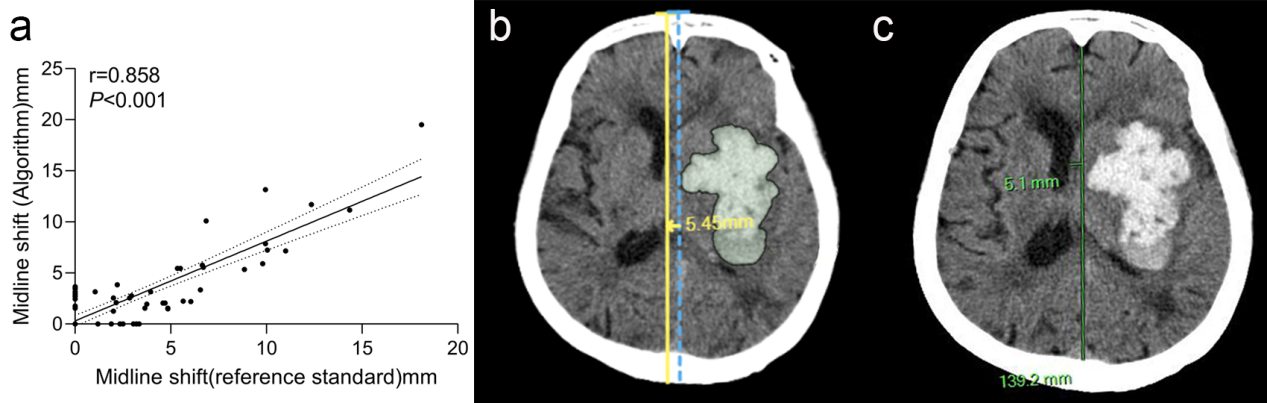
Fig. S4 a, Scatter plots comparing measured spontaneous supratentorial intracerebral hemorrhage (ICH) midline shift between the algorithm measurement and reference standard. b-c, Examples of ICH midline shift measurement. The midline shift was 5.4mm for algorithm measurement (b) and 5.1mm for reference standard (c).
